# Perivascular epithelioid cell tumors (PEComa) of the female genital tract: A challenging question for gynaecologic oncologist and pathologist

**DOI:** 10.1016/j.gore.2020.100603

**Published:** 2020-07-06

**Authors:** Angiolo Gadducci, Gian Franco Zannoni

**Affiliations:** a Department of Clinical and Experimental Medicine, Division of Gynecology and Obstetrics, University of Pisa, Italy; b Division of Anatomic Pathology and Histology – Fondazione Policlinico Universitario A. Gemelli IRCCS, Università Cattolica del Sacro Cuore School of Medicine, Rome, Italy

**Keywords:** Uterine perivascular epithelioid cell tumors (PEComas), Cervical PEComas, Ovarian PEComas, Melanocytic markers, Myogenic markers

## Abstract

Perivascular epithelioid cell tumors (PEComa)s are mesenchymal neoplasms composed of perivascular epithelioid cells, which express both melanocytic and myogenic markers. These neoplastic cells are thought to arise from undifferentiated cells of the neural crest, or from myoblastic cells harbouring a molecular alteration leading to expression of melanocytic markers, or from pericytic cells. The majority of cases are benign, but a small subset behaves in a malignant fashion. Unfortunately, given their rarity, reliable criteria for predicting malignancy have yet to be established. PEComas of the female genital tract account for nearly 25% of PEComas of all body sites, and the most common site of occurrence is the uterine corpus; less common sites include the cervix, adnexa, vagina/vulva or broad or round ligament exceptionally. The present review aims to elucidate the clinical, pathological and molecular features of gynecological PEComas. Moreover, prognostic and therapeutic implications are also discussed.

## Highlights

• Perivascular epithelioid cell tumors (PEComa)s are mesenchymal neoplasms expressing both melanocytic and myogenic markers.
• Gynecological PEComas account for nearly 25% of all PEComas, and the most common site of occurrence is the uterine cavity.
• These tumors are quite always diagnosed postoperatively by definitive histological examination.
• Complete surgical resection with a tumor-free margin is usually considered to be the standard treatment.

## 1 Introduction

The term perivascular epithelioid cell tumors [PEComa]s refers to a family of mesenchymal neoplasms composed of characteristic cells which usually express both melanocytic and myogenic markers such as human melanoma black [HMB] 45, human melanosome-associated antigen-1 [HMSA-1], MelanA/Mart1, microphthalmia transcription factor [MiTF], smooth muscle actin [SMA], pan-muscle actin, muscle myosin, calponin, sometimes h-caldesmon and, less commonly, desmin (Bonetti et al., 1997, Thway and Fisher, 2015). The cells in PEComas are arranged around blood vessels and seems to form the vessel wall, often infiltrating the smooth muscle of small- to medium-sized vessels (Thway and Fisher, 2015). The cells have small, round to oval nuclei, with inconspicuous nucleoli, sometimes with focal nuclear atypia, and clear to eosinophilic cytoplasm. No counterpart normal cell has been detected, and it has been hypothesized that PEComas arise from: i) undifferentiated cells of the neural crest expressing both smooth muscle and melanocytic phenotype, or ii) myoblastic cells harbouring a molecular alteration leading to expression of melanocytic markers, or iii) pericytic cells (Thway and Fisher, 2015).

The PEComa family includes angiomyolipoma, pulmonary clear cell “sugar” tumor, lymphangioleiomyomatosis, primary extrapulmonary sugar tumor, clear cell myomelanocytic tumor of the falciform ligament/ligamentum teres, abdominopelvic sarcoma of perivascular epithelioid cells, and other similar tumors in different sites (Thway and Fisher, 2015, Folpe et al., 2000, Tazelaar et al., 2001).

PEComas can be related to genetic alterations of tuberous sclerosis complex [TSC], an autosomal dominant disease due to loss of TSC1 or TSC2 genes involved in the regulation of the Phosphatidylinositol 3-Kinases [PI3K]/AKT/Mammalian Target of Rapamycin [mTOR] signaling pathway (Kwiatkowski, 2003). TSC gene alterations have been detected in several PEComas, occurring both within the TSC and in sporadic cases. The pathophysiology of aberrant mTOR signaling offers a strong rationale to target this pathway and structurally similar mTOR inhibitors, such as sirolimus or everolimus, have shown promising therapeutic activity in PEComas of different sites (Bissler et al., 2008, Wagner et al., 2010, Gennatas and Michalaki, 2012).

A distinct subset of PEComas harbouring fusions of transcription factor 3 [FT3] gene (member of the MiFT family) is characterized by young age, absence of the association with TSC, predominant alveolar architecture and epithelioid cytology, and minimal immunoreactivity for muscle markers (Rao et al., 2013, Malinowska et al., 2012, Agaram et al., 2015). Combined RNA sequencing and fluorescence in situ hybridization [FISH] of 38 PEComas of different sites detected 9 (23%) TFE3 gene-rearranged tumors (Agaram et al., 2015). TSC2 mutations were found in 80% of TFE3 fusion-negative cases and concomitant p53 mutations were noted in 63% of TSC2-mutated cases, which confirmed the hypothesis that different molecular pathways could be involved in the pathogenesis of PEComas. These tumors may arise from multiple anatomical sites, including the kidney, lung, bladder, prostate, pancreas, liver, falciform ligament/ ligamentum teres, breast, skin, eye, skull base, colon, soft tissues, and more frequently from retroperitoneum and female genital tract (Folpe et al., 2000, Tazelaar et al., 2001, Folpe et al., 2005, Fadare, 2008, Conlon et al., 2015, Acosta, 2017, Bennett et al., 2018, Liu et al., 2019, Bleeker et al., 2012).

Folpe et al. (Folpe et al., 2005), who reassessed 26 PEComas of soft tissue and gynecologic origin, suggested to classify these tumors as benign, of uncertain malignant potential, or malignant, on the basis of six worrisome findings (Table 1). In their series, local or distant failure was significantly related to tumor size (>8cm), mitotic activity (>1/50 HPF) and necrosis

**Table 1:** Worrisome features predictive of outcome in patients with PEComas.

Folpe et al. (12)		Schoolmeester et al.(19)		Bennett et al (16)	
category	Histologic criteria	Category	Histologic criteria	Category	Histologic criteria
Benign	**No worrisome features:** less than5 cm in diameter Non-high nuclear grade and cellularity Mitotic rate ≤ 1/50 HPF No necrosis No vascular invasion	**Benign/uncertain malignant potential**	**Less than 4 worrisome features:** size ≥ 5 cm high-grade atypia (excluding degenerative atypia) mitoses > 1/50 HPF, necrosis lymphovascular invasion	**Uncertain malignant potential**	**Less than 3 worrisome features:** size ≥ 5 cm high-grade atypia (excluding degenerative atypia) mitoses > 1/50 HPF, necrosis lymphovascular invasion
Uncertain malignant potential	**One or both of the following features:** Nuclear pleomorphism/multinucleated giant cells only or size>5 cm	**Malignant**	**4 or more worrisome features:** size ≥ 5 cm, high-grade atypia (excluding degenerative atypia) mitoses > 1/50 HPF, necrosis lymphovascular invasion	**Malignant**	**3 or more worrisome features:** size ≥ 5 cm, high-grade atypia (excluding degenerative atypia) mitoses > 1/50 HPF, necrosis lymphovascular invasion
Malignant	**Two or more worrisome features:** >5 cm in diameter Infiltrative High nuclear grade and cellularity Mitotic rate > 1/50 HPF Necrosis Vascular invasion				

Legend: HPF: high power field

Bleeker et al (Bleeker et al., 2012), who evaluated 234 PEComas of different sites, found that tumor size ≥ 5 cm (p = 0.02) and high (>1/50 HPF) mitotic rate (p less than 0.001) were the only variables predictive of failure after surgery.

## 2 Gynecologic PEComa

### 2.1 Incidence and general findings

PEComas of the female genital tract account for nearly 25% of all PEComas of all body sites e, and the original site of the tumor is the uterine corpus in most cases (Folpe et al., 2005, Fadare, 2008, Conlon et al., 2015, Acosta, 2017, Bennett et al., 2018, Liu et al., 2019, Schoolmeester et al., 2014, Fukunaga, 2005, Liu et al., 2009, Musella et al., 2015, Choi et al., 2016, Kwon et al., 2017, Shan et al., 2019, Nishio et al., 2019), the cervix rarely (Wagner et al., 2010, Folpe et al., 2005, Conlon et al., 2015, Liu et al., 2019, Bradshaw et al., 2010, Yamamoto et al., 2010, Stone et al., 2013, Natella et al., 2014, Kudela et al., 2016, Kovac et al., 2018), and adnexa (Conlon et al., 2015, Liu et al., 2019, Schoolmeester et al., 2014, Anderson et al., 2002, Ramaiah et al., 2009, Westaby et al., 2017, Rampisela et al., 2016), vagina/vulva (Tazelaar et al., 2001, Folpe et al., 2005, Conlon et al., 2015, Liu et al., 2019, Schoolmeester et al., 2014, Kalyanasundaram et al., 2005, Ong et al., 2007, Schoolmeester et al., 2015, Cho et al., 2014) or broad or round ligament (Rao et al., 2013, Folpe et al., 2005, Conlon et al., 2015, Fink et al., 2004, Kim et al., 2006, Phongnarisorn et al., 2009, Staley et al., 2015) exceptionally.

Liu et al. (Liu et al., 2019), who retrospectively assessed 114 cases of gynecological PEComas published in the English literature between 1997 and 2017, reported that the tumor involved the uterine body in 82 cases (71.9%), cervix in 12 (10.5%), adnexa in 7 (6.1%), vagina in 7 (6.1%), broad ligament in 5 (4.4%), and vulva in one case (0.9%). Patient age ranged from 6 to 80 years, with a median age of 49 years for uterine tumors, 46 years for cervical tumors, 50 years for adnexal tumors, 28 years for vaginal PEComas, 25 years for broad ligament tumors, and 20 years for vulvar tumor.

### 2.2 Pathologic features

#### 2.2.1 Gross examination

PEComas are usually well circumscribed lesions of soft consistency, without a definite capsule (Thway and Fisher, 2015). The reported tumor diameter ranges from 0.2 to 17 cm. On gross examination, most tumors show a tan to gray to yellow–brown to white cut surface, with focal areas of necrosis or hemorrhage (Bennett et al., 2018).

#### 2.2.2 Microscopic examination

**Fig. 1:**
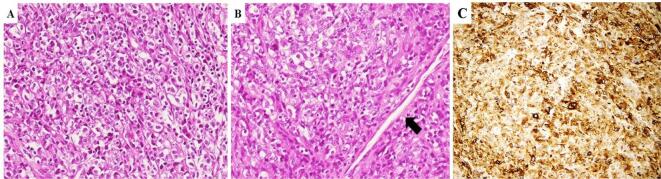
Haematoxylin and eosin (H&E) stained sections illustrating the histopathological features of uterine PEComa A). Neoplastic cells are arranged in nested pattern and show abundant clear and granular cytoplasm with small, round to oval nuclei and small nucleoli. B). This tumor exhibits a useful diagnostic clue: neoplastic cells are arranged around a vascular space (arrow). C). Diffuse immunohistochemical expression for MELAN-A is depicted.

Histologically, two main morphologic subtypes of gynecologic PEComas have been described (Folpe et al., 2005, Conlon et al., 2015, Bennett et al., 2018). The first one displays finger-like permeation of the uterine wall and is closely reminiscent of low-grade endometrial stromal sarcoma. Neoplastic cells show abundant eosinophilic, clear, or granular cytoplasm with small, round to oval nuclei, small nucleoli, slight atypia, and low mitotic activity (Fig. 1). These tumors exhibit strong HMB-45 expression with only focal smooth-muscle marker positivity (Folpe et al., 2005, Conlon et al., 2015, Bennett et al., 2018). The other subtype shows overlapping morphological and immunohistochemical features with epithelioid smooth muscle tumors. This PEComa subtype is composed of epithelioid cells with less prominent clear cell morphology, less HMB-45 expression, and more extensive smooth-muscle marker positivity (Folpe et al., 2005, Conlon et al., 2015, Bennett et al., 2018).

The neoplastic cells in both subtypes are usually arranged in nested pattern; other less common patterns include sheets, trabeculae, cords, fascicles, single cells, and pseudo-alveoli (Bonetti et al., 1997, Thway and Fisher, 2015, Folpe et al., 2005, Conlon et al., 2015, Bennett et al., 2018).

**Fig. 2:**
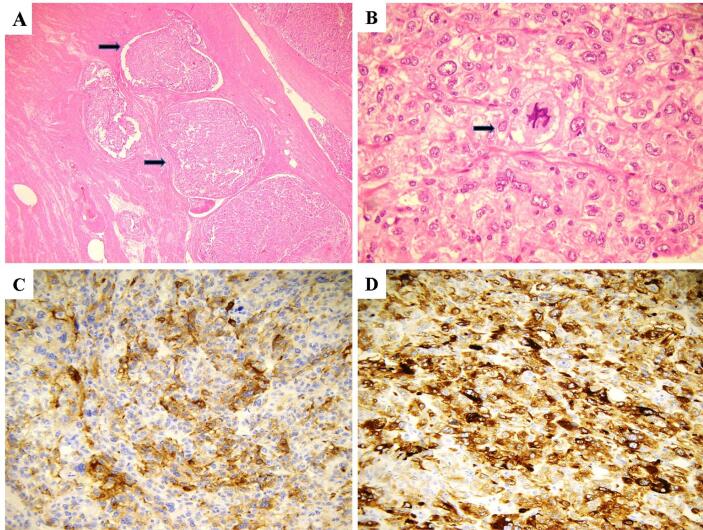
Haematoxylin and eosin (H&E) stained sections illustrating the histopathological features of malignant uterine PEComa. A). The present case showed infiltrative growth pattern and extensive lympho-vasular invasion (arrows). B). This tumor also exhibited significant cytological atypia and increased mitotic activity also with atypical mitotic figures (arrow). C). Immunohistochemical expression for HMB-45 is depicted. D). Neoplastic cells were also positive for MELAN-A.

An useful diagnostic clue is represented by the presence of areas where neoplastic cells are arranged around vascular spaces. Less common histological findings include: stromal hyalinization, rhabdoid cells, multinucleated cells, Touton giant cells, intranuclear pseudoinclusions and melanin pigment (Folpe et al., 2005, Conlon et al., 2015, Bennett et al., 2018). Fig. 2 shows the histopathological features of malignant uterine PEComa.

#### 2.2.3 Histological parameters predictive of aggressive clinical behaviour

The histologic features usually related to aggressive clinical behaviour include the following: tumor size > 5 cm; infiltrative growth pattern; high nuclear grade; necrosis; and > 1 mitosis/50 HPF (Folpe et al., 2005, Conlon et al., 2015, Bennett et al., 2018). Based on these findings, Folpe et al. (Folpe et al., 2005) suggested that small PEComas without any worrisome features are most likely benign, PEComas with nuclear pleomorphism alone (“symplastic”) and large PEComas without other worrisome features have uncertain malignant potential, and PEComas with two or more worrisome features are considered malignant. (Table 1)

Using these criteria for their series of 16 gynecologic PEComas, Schoolmeester et al. (Schoolmeester et al., 2014) noted that not only all aggressive PEComas but also 57% of PEComas with favorable outcome were classified as malignant. Therefore, these authors suggested a modified algorithm based on five worrisome features, with the presence of at least four features required for a diagnosis of malignant PEComa (Table 1). All their 16 tumors were correctly classified with this novel algorithm, which reduced the number of categories to two—benign/uncertain malignant potential and malignant. Bennet et al (Bennett et al., 2018) evaluated the morphologic, immunohistochemical, and molecular findings of 32 uterine PEComas, including 11 with aggressive behavior, and found that Schoolmeester et al.’ criteria (Schoolmeester et al., 2014) incorrectly classified 36% (4/11) of aggressive tumors. These authors proposed a modified algorithm with a threshold of three worrisome features required to classify a PEComa as malignant. Moreover, they eliminated the term “benign” in the benign/uncertain malignant potential category (Table 1). Malignant PEComas can spread to the vagina, fallopian tubes, ovaries, bladder, and ureters and can metastasize to lungs, and, less frequently, to liver, bowel, lymph nodes and peritoneal cavity (Wagner et al., 2010, Kwon et al., 2017, Shan et al., 2019, Yoo-Bee et al., 2016, Fukunaga, 2005, Liu et al., 2009, Musella et al., 2015).

#### 2.2.4 Immunohistochemical and molecular features

PEComas are characterized by immunoexpression of both myoid (desmin, SMA, muscle-specific-actin, muscle myosin, and calponin) and melanocytic (HMB-45, Melan-A/MART-1, tyrosinase, and MiTF) markers (Folpe et al., 2005, Conlon et al., 2015, Bennett et al., 2018, Bonetti et al., 1997, Thway and Fisher, 2015). Cathepsin K represents another useful diagnostic immunomarker which is frequently and strongly expressed in PEComas (Rao et al., 2013). Moreover, hormone receptors may play a role in the pathogenesis of gynecologic PEComas. In fact, estrogen and progesterone receptors positivity has been observed in the spindle cell component of uterine PEComas. Sporadic cases showing CD117 expression have also been reported (Folpe et al., 2005, Conlon et al., 2015, Bennett et al., 2018).

From a molecular perspective, these tumors are well known to occur in the setting of TSC; however, even in sporadic cases, the most frequent genetic alterations regard the loss of heterozygosity of the TSC2 gene (16p13.3) or, less commonly, TSC1 (9q34). A small subset of PEComas shows rearrangement of the TFE3 gene (Malinowska et al., 2012, Agaram et al., 2015). These tumors are usually composed of epithelioid cells with clear cytoplasm and nested architecture and lack immunohistochemical expression of SMA and desmin.

#### 2.2.5 Pathological differential diagnosis

The pathological differential diagnosis of uterine PEComas is broad and include all mesenchymal neoplasms showing spindled and/or epithelioid cells features. The main neoplasms to be considered in the differential diagnosis include: smooth muscle tumors, endometrial stromal sarcomas and metastatic malignant melanomas (Folpe et al., 2005, Conlon et al., 2015, Bennett et al., 2018).

Smooth muscle tumors, especially with epithelioid morphology, show significant morphological overlap with PEComas. The main features favouring the latter include: eosinophilic to clear cytoplasm; round to oval nuclei; prominent capillary network; extensive staining for Melan-A, HMB-45, MITF and Cathepsin K. Moreover EMA and cytokeratins staining are observed in epithelioid smooth muscle tumors but not in Pecomas (Folpe et al., 2005, Conlon et al., 2015, Bennett et al., 2018).

Endometrial stromal sarcomas may also resemble PEComas, especially epithelioid cell variants, since both neoplasms can exhibit similar infiltrative myometrial pattern. However, endometrial stromal sarcomas show morphological evidence of endometrial involvement and endometrial stromal differentiation; furthermore, their immunoistochemical profile, CD10+ /HMB-45, Melan-A-; MiTF, should help in the distinction from PEComas (Folpe et al., 2005, Conlon et al., 2015, Bennett et al., 2018).

Lastly, the possibility of metastatic malignant melanoma can also be ruled out by obtaining clinical information regarding previous history of melanocytic lesions and by immunoistochemistry. In fact, PEComas usually express smooth muscle markers which are never observed in melanomas (Folpe et al., 2005, Conlon et al., 2015, Bennett et al., 2018).

### 2.3 Clinical presentation and imaging examinations

Patients usually experience vaginal bleeding, abdominal pain or discomfort or enlargement and palpable abdominal mass, ad rarely rupture of the uterus and hemoperitoneum (Liu et al., 2019, Musella et al., 2015, Kovac et al., 2018, Cho et al., 2014, Staley et al., 2015, Rothenberger et al., 2019). Rothenberger et al. (Rothenberger et al., 2019) reported a case of uterine PEComa in a 61-year old woman presenting with diffuse petechaie, mucosal and vaginal bleeding, severe thrombocytopenia and disseminated intravascular coagulation. After emergency treatment of coagulopathy, an abdominal- pelvic CT scan detected an apparently large uterine fibroid and an endometrial biopsy revealed an uterine PEComa. A 44-year-old woman presented with pneumothorax due to lung metastases from a malignant uterine PEComa (Okamoto et al., 2017).

Some women have concomitant uterine disease at presentation, such as endometrial carcinoma and leiomyoma (Choi et al., 2016). Kwon et al. (Kwon et al., 2017) described a case of synchronous uterine PEComa with lymph node involvement detected occasionally after staging surgery for ovarian cancer in a 38-year-old woman with a history of TSC. Definitive diagnosis emerged only at the histologic examination of hysterectomy specimen.

On clinical examination, cervical PEComa can appear as a friable, solid mass (Bradshaw et al., 2010) or a polypoid lesion.

Only anecdotal data are currently available as for PAP smear in cervical PEComas. Stone et al. (2013) noted an uniform population of discohesive cells with a fragile, pale cytoplasm, uniform nuclei with finely stippled chromatin and a distinct single prominent nucleolus, suggestive of a possible high-grade glandular lesion with a more specific diagnosis of clear cell carcinoma. A cone biopsy revealed a 9‐mm nodule, not appreciated on colposcopy, which was correctly diagnosed at microscopic and immunohistochemical examination.

At ultrasound examination an uterine PEComa can show either a heterogeneous echotexture with no cystic areas or significant vascularity on Doppler examination and with well defined margins, similarly to a fibroid, or a hyperechogeneous aspect with no clear separation from the adjacent uterus and an extremely rich central vascular network, similarly to a leiomyosarcoma (Socolov et al., 2016). Magnetic resonance [MR] imaging can better define the internal structure of the lesion, but a wide range of imaging characteristics have been reported (Kwon et al., 2017, Nishio et al., 2019, Yoo-Bee et al., 2016). MR can display a heterogeneous mass which is hypointense on T1-weighted image and isointense or hyperintense on T2- weighted image, with significant enhancement uptake after gadolinium.Pre-contrast non-fat-suppressed and post-gadolinium fat-suppressed T1-weighted MR images can demonstrate homogeneous isointense signal of mass with heterogeneous enhancement. At MR examination an uterine PEComa can appear as a well- circumscribed homogeneous submucosal mass with signal intensity or enhancement similar to that of the myometrium (Kwon et al., 2017), or as a well- defined uterine wall tumor mimicking a swornstorm and avidly capturing gadolinium, or as single or multiple, irregularly shaped or lobulated hemorrhagic lesions within the myometrium that differ from typical adenomyotic cysts in their larger size and irregular margins (Nishio et al., 2019). Malignant PEComas are hypodense or isodense on unenhanced CT scan, but usually present significant homogeneous or heterogeneous enhancement after contrast medium. A mixed PEComa and mesonephric adenocarcinoma of the cervix appeared as a hypoechoic mass in the proximal endocervix with some internal color flow Doppler signals at ultrasound, and as an endocervical mass with an intermediate signal intensity at MR imaging.

In conclusion, an accurate pre-operative assessment of PEComa is almost impossible based on imaging features alone, and these tumors are quite always diagnosed postoperatively (Kwon et al., 2017, Shan et al., 2019). CT scan and 18 fluorodeoxyglucose [FDG]^18^ positron emission tomography [PET]/CT may be useful for the detection of unsuspected metastases, the assessment of response to treatment, and follow-up of patients with malignant PEComas, but the literature data on these issues are very scanty (Yoo-Bee et al., 2016, Ciarallo et al., 2011).

### 2.4 Treatment

Although there is no consensus regarding the management of gynecological PEComas owing to their rarity, complete surgical resection with a tumor-free margin is usually considered to be the standard treatment (Liu et al., 2019, Musella et al., 2015, Kwon et al., 2017, Shan et al., 2019, Kudela et al., 2016, Kovac et al., 2018, Fink et al., 2004), whereas only anecdotal and inconclusive data are available as for adjuvant radiotherapy (Fink et al., 2004) or chemotherapy (Shan et al., 2019). These tumors tend to recur both locally and in distant sites, especially in the lung, even many years after initial surgery (Greene et al., 2003, Armah and Parwani, 2007). Several chemotherapeutic drugs, such as dacarbazine, ifosfamide, doxorubicin, vincristine, cyclophosphamide, irinotecan, and paclitaxel, have been tested in advanced, metastatic or recurrent PEComas of different sites with contrasting results (Agaram et al., 2015, Liu et al., 2009, Kalyanasundaram et al., 2005, Ong et al., 2007, Jeon and Lee, 2005). A 9-year-old girl with metastatic uterine PEComa received a combination of chemotherapy with vincristine, ifosfamide, and doxorubicin plus radiotherapy after surgery and she was disease-free 1.5 years after diagnosis (Jeon and Lee, 2005). A 33-year old woman with an uterine PEComa and lymph node mestastases underwent neoadjuvant chemotherapy, debulking surgery and adjuvant chemotherapy and was free of disease 8 months after surgery (Liu et al., 2009). A 16-year-old girl with a vaginal PEComa recurred 10 months after resection and chemotherapy (Kalyanasundaram et al., 2005).

mTOR inhibitors have shown antineoplastic activity in PEComas of different sites, including the uterus (Liu et al., 2019, Italiano et al., 2010, Starbuck et al., 2016, Bissler et al., 2008, Wagner et al., 2010, Gennatas and Michalaki, 2012). However these drugs act as cytostatic agents able to induce cell cycle arrest rather than cell death and the disease typically regrows after drug discontinuation (Bissler et al., 2008). Sirolimus obtained only a short- lived reduction in size and central cavitation of most pulmonary metastases in a woman with uterine PEComa. (Wagner et al., 2010).Three patients with advanced uterine PEComas underwent debulking surgery followed by mTOR inhibitors: two of these obtained a complete response and the remaining patient developed progressive disease (Starbuck et al., 2016). Temsirolimus was administered to two patients with pulmonary metastases from uterine PEComa (Italiano et al., 2010). One patient a obtained PET/CT proven complete response followed by surgery, but the pathologic examination of the surgical sample revealed residual viable tumor cells with a reduced mitotic activity compared with primary uterine PEComa. She received mTOR inhibitor as consolidation treatment and was free of disease 9 months later. The second patient achieved an initial partial response but progressed 22 weeks after initiation of temsirolimus.

The response of TFE3 gene-rearranged PEComas to mTOR inhibitors is not well defined, and. other molecular targeted agents, such as the MET inhibitor crizotinib, can be tested in these malignancies (Agaram et al., 2015). An European Organization for Research and Treatment of Cancer [EORTC] phase II trial has recently shown that crizotinib is active in patients with alveolar soft part sarcoma with TF3 rearrangement (Schöffski et al., 2018).

### 2.5 Prognosis

Follow-up data were available for 68 of the 82 patients with uterine PEComa reassessed by Liu et al. (2019). After a median follow-up of 19 months, 47 (69.1%) were alive with no evidence of disease, 11 (16.2%) died of disease, and the remaining were alive with disease. Additional 11 cases of uterine PECOmas were reported between 2018 and 2019, and the clinical status was known for 8 of these: 7 were free of disease after a median interval of 22 months (range, 3 to 71 months) and one died of disease 10 months after surgery (Shan et al., 2019, Nishio et al., 2019, Rothenberger et al., 2019).

Fifteen cases of cervical PEComas have described in the literature, and their surgical approach ranged from tumor resection to total hysterectomy or radical hysterectomy with bilateral salpingectomy-oophorectomy and pelvic lymphadenectomy to pelvic exenteration (Wagner et al., 2010, Folpe et al., 2005, Conlon et al., 2015, Liu et al., 2019, Bradshaw et al., 2010, Yamamoto et al., 2010, Stone et al., 2013, Natella et al., 2014, Kudela et al., 2016, Kovac et al., 2018). The clinical status is known for 11 of these.Two patients treated with tumor resection developed recurrent disease in the cervix after four months (Yamamoto et al., 2010). The former underwent tumor re-excision, and a further resection for a second recurrence was performed seven months later. She was free of disease 12 months after the last operation. The other patient was re-excised and was alive with suspicious metastatic pelvic lymph nodes by imaging studies 5 months after the first operation. Of the 8 patients who underwent total or radical hysterectomy (followed by radiotherapy in one case), 7 patients were free of disease after a median follow- up of 36 months (range,12–42 months), and one patient with lung metastases at presentation received sirolimus after surgery and died approximately 9 months later (Wagner et al., 2010, Folpe et al., 2005, Bradshaw et al., 2010, Kovac et al., 2018).

A 52 year- old woman with a huge malignant PEComa arising from the cervix and vagina failed to respond to radiotherapy and then underwent a pelvic exenteration (Natella et al., 2014). The tumor infiltrated the cervix, the vagina, the bladder and the serosa of the rectum, with no evidence of lymph nodal or metastatic disease. She received no additional treatment and was free of disease after 12 months.

Adnexal PEComas are exceptionally rare, with only 5 primary cases and 7 cases metastatic to the ovaries reported in the literature (Conlon et al., 2015, Liu et al., 2019, Schoolmeester et al., 2014, Anderson et al., 2002, Ramaiah et al., 2009, Westaby et al., 2017, Rampisela et al., 2016, Bonetti et al., 2001, Yanai et al., 2003, Froio et al., 2008, SX Liang et al., 2008). Ramaiah et al. (Ramaiah et al., 2009) reported a case of sclerosing adnexal malignant PEComa in a 63-year- old woman who underwent total hysterectomy, bilateral salpingo-oophorectomy, omental biopsy and left pelvic lymph node biopsy for a huge mass in the left pelvis associated with a solitary hypoechoic suspected hepatic lesion. At microscopic examination, the left ovary and fallopian tube were completely destroyed by PEComa, and the excised lymph node contained a high- grade metastatic tumor with extranodal extension. The patient early developed lung and liver progression and died 4 months after surgery.

Han Yoo-Bee et al (Yoo-Bee et al., 2016) described the case of a 48-year-old, previously hysterectomized woman who underwent bilateral salpingo-oophorectomy and wedge resection of the lung for a PEComa of the ovaries with pulmonary metastases, but follow-up information were not available. A 3.0 × 2.5 cm ovarian PEComa was incidentally found in the left ovary of a 43-year-old black woman who was free of tumor 7 years after surgery (Rampisela et al., 2016).

PEComas arising from the vagina, vulva, broad ligament and round ligament have been reported in 8 cases (Folpe et al., 2005, Schoolmeester et al., 2014, Kalyanasundaram et al., 2005, Ong et al., 2007, Schoolmeester et al., 2015, Cho et al., 2014), one case (Tazelaar et al., 2001), 5 cases (Rao et al., 2013, Folpe et al., 2005, Fink et al., 2004, Kim et al., 2006, Staley et al., 2015) and 2 cases (Phongnarisorn et al., 2009, Nitahara et al., 2019), respectively.

Three patients with PEComas of the vagina were free of disease after a median time of 12 months (range, 3–17 months) from resection (Schoolmeester et al., 2015, Cho et al., 2014), whereas another patient with this tumor recurred 10 months after resection and chemotherapy as previously reported (Kalyanasundaram et al., 2005). Ong et al. (Ong et al., 2007) described the case of a 8-year old girl in whom a vaginal lesion was diagnosed at initial biopsy as embryonal rhabdomyosarcoma. After three ineffective cycles of chemotherapy with ifosfamide, vincristine, and actinomycin D, the patient underwent partial vaginectomy with total resection of the tumor and the histological examination of the surgical specimen revealed a PEComa. She was free of disease 6 months later.

Tazelar et al. (Tazelaar et al., 2001) reported a case of vulvar PEComa with low mitotic count and no nuclear atypia in a 20-year woman who was free of disease after 48 months.

As for the patients with PEComas of the broad ligament, a 51-year-old woman treated with total hysterectomy, bilateral salpingo-oophorectomy, omentectomy and pelvic radiotherapy was free of disease 15 months after diagnosis (Fink et al., 2004), a 33-year old woman with a mass compressing / infiltrating distal left ureter was free of disease 24 months after tumor excision and ureteric reimplantation (Nitahara et al., 2019), whereas a 12- year old girl recurred in the form of multiple masses in the right iliac fossa within 12 months from surgery (Kim et al., 2006). A 44-year-old patient presenting with hemoperitoneum underwent subtotal hysterectomy, bilateral salpingo-oophorectomy and excision of the broad ligament mass, but no follow-up data were given (Staley et al., 2015). A 45-year-old woman was free of disease 18 months after laparoscopic radical excision of primary round ligament PEComa mimicking a leiomyoma (Phongnarisorn et al., 2009)

## 3 Conclusions

PEComas are very rare tumors of the female genital tract, that usually arise from the uterine body. Symptoms and signs are not specific and the diagnosis emerges from an accurate histologic and immunohistochemical study of the surgical specimens, whereas the preoperative imaging examinations are unable to discriminate these tumors from other benign or malignant gynecological conditions such as fibroid or leiomyosarcoma. The treatment of choice is represented by the complete surgical resection with tumor free margins, whereas there are no data as for adjuvant radiotherapy or chemotherapy. From a theoretic point of view radiotherapy could be useful for a better local control of malignant PEComas with high mitotic count and rich vascularization but its role is unproven (Musella et al., 2015). On the other hand, the rarity of these tumors does not allow the performance of randomized controlled trials comparing different therapeutic approaches

The prognosis is usually favorable, the majority of the patients reported in the studies are free of disease and only a few die of tumor (Liu et al., 2019). A long-term surveillance program is anyway recommended, especially for patients with high risk-features, and the use of mTOR inhibitors should be taken into consideration in patients with residual disease after surgery and well as in those with recurrent or metastatic disease (Wagner et al., 2010, Liu et al., 2019, Italiano et al., 2010, Starbuck et al., 2016).

## CRediT authorship contribution statement

**Angiolo Gadducci:** Conceptualization, Writing - original draft, Data curation, Formal analysis, Methodology, Writing - review & editing. **Gian Franco Zannoni:** Data curation, Formal analysis, Methodology, Writing - review & editing.
